# A protocol using mixed methods for the impact analysis of the implementation of the EMPOWER project: an eHealth intervention to promote mental health and well-being in European workplaces

**DOI:** 10.1136/bmjopen-2023-082219

**Published:** 2025-04-09

**Authors:** Luis Salvador-Carulla, Sue Lukersmith, Cindy E Woods, Tom Chen, Carlota de Miquel

**Affiliations:** 1 Health Research Institute, University of Canberra Faculty of Health, Bruce, Australian Capital Territory, Australia; 2 Health Research Institute, University of Canberra Faculty of Health, Canberra, Australian Capital Territory, Australia; 3 Canberra Business School, University of Canberra Faculty of Business Government & Law, Canberra, Australian Capital Territory, Australia; 4 Parc Sanitari Sant Joan de Deu Xarxa de Salut Mental, Barcelona, Spain

**Keywords:** MENTAL HEALTH, Implementation Science, Occupational Stress, Behavior

## Abstract

**Abstract:**

**Introduction:**

Mental health at the workplace has been identified as a major priority by the World Health Organization (WHO). Despite its significance, international studies examining the influence of digital mental health interventions on workplace implementation and outcomes are lacking. The European Platform to Promote Well-being and Health in the Workplace (EMPOWER) platform is an eHealth intervention consisting of a website and web-based app designed to guide employers and employees on the prevention of common health problems, reduce presenteeism and absenteeism in the workplace. The aim of this paper is to describe the rationale and methods that will be used to conduct a maxi impact analysis of the processes undertaken to develop and implement the EMPOWER platform in European workplaces using the Global Impact Analytics Framework (GIAF) methodology.

**Methods and analysis:**

We will undertake a mixed-methods analysis of the impact of the process of implementation in the two phases of implementation (initiation and maturity—the early implementation phase). The primary methodology that will be used for the analysis is the GIAF and toolkit. The GIAF toolkit includes a taxonomy (knowledge map), glossary and checklists to examine and rate the EMPOWER project across various domains of impact: planning, pre-engagement, readiness, usability, dissemination, adoption and uptake. Information will be collected from a range of sources through different methods and used to rate the EMPOWER platform (website and app) on each domain. For reliability and validity, four raters will independently rate the EMPOWER platform using the same information. The analysis will include qualitative and quantitative methods to rate on standardised ladders and scales in the GIAF toolkit. Analysis will include descriptive statistics and non-parametric tests where relevant. The information gained will be reviewed in a subgroup (per country) and group (three country) analysis for formative and key summative learnings. These key learnings will be synthesised to generate organisational learnings and insights for the EMPOWER consortium to improve future intervention implementation processes.

**Ethics and dissemination:**

The impact analysis study protocol has been approved by the Research Ethics Committees of the University of Canberra (ID:202311841) and also the Fundació Sant Joan de Déu (PIC-39-20). The participating countries for the RCT (EMPOWER study) also obtained ethical approval through their respective ethical organisations in the participating countries. The impact analysis is registered with the Open Science Framework ID osf.io/eysc9. The EMPOWER project trial is registered at ClinicalTrial.gov with trial ID NCT04907604. The outcomes of the impact analysis study will be disseminated via conference presentations, peer-reviewed journals and key organisational learnings presented in relevant forums.

**Trial registration number:**

NCT04907604.

**STRENGTHS AND LIMITATIONS OF THIS STUDY**

- A limitation of the study is the limited opportunities to inform and educate consortium partners on the Global Impact Analytics Framework (GIAF) ecosystem approach to impact analysis.
- A further limitation of this study is the inherent complexity and need to rely on third-party data collection of dissemination activities in the study sites (workplaces) in real time.
- A strength of this study is the use of the GIAF which accommodates an International multiproject maxi impact assessment.
- A further strength is the GIAF framework, which provides a methodology for a rigorous evaluation, which focuses on the pathways to develop the European Platform to Promote Well-being and Health in the Workplace platform and then the process of its implementation in real-world workplaces.

## Introduction

 Mental health at the workplace has been identified as a major priority by the WHO.^1^ Despite its significance, international studies examining the impact of digital mental health interventions implemented in the workplace are limited, with the focus on tools implemented in healthcare organisations.^2^,^4^ Inadequate evaluation and documentation of the process of implementation make it challenging for those implementing new digital interventions to replicate positive results or overcome barriers encountered in similar contexts.^5 6^ This is due to a range of factors including: inherent challenges posed by the different designs and methods in digital health research from the methods developed to test real-world applications; the rapid cycle of innovation in the digital sector; the inadequate time frame of funding of research projects (typically 4 years) to assess implementation; its cross-disciplinarity and differences in reporting methods.^7 8^

Another relevant barrier to evaluation is the absence of an international consensus on the terminology and classification (taxonomy) in implementation sciences. As an example, ‘Reach’, ‘Effectiveness’, ‘Adaptation’, ‘Implementation’ and ‘Maintenance’ are the domains of one of the main frameworks used in impact analysis,^9^ but the definitions and use of these terms vary widely among the research groups working in implementation.^9^,^11^ Terminology is defined as ‘a set of designations belonging to one special language’.^12^ Its purpose is to eliminate ambiguity from scientific language by means of standardisation.^12 13^

Added to the lack of a common taxonomy and its nomenclature, there is a dearth of standardised tools for quantitative impact analysis of the process of implementation.^14^,^16^ Previous work on impact analysis conducted by our group identified existing gaps in the evaluation of the process of implementation.^17^ Filling this gap is critical to set up inferences linking the inputs (resources) (eg, capacity and other characteristics of the ‘sender’ and the ‘recipients’ of an application), to the final research outputs (results) by providing analysis tools that shed light on the process (throughputs) of implementation.^17 18^

The Global Impact Analytics Framework (GIAF) is a novel methodology for impact analysis developed by an Australian-based team in partnership with a consortium of international topic experts. The GIAF uses an ontoterminology approach by including a taxonomy of the process of implementation, an accompanying glossary of terms and a series of checklists to evaluate the different domains of the taxonomy. The methodology uses qualitative and quantitative analysis. Through the use of the GIAF, there is also the potential to identify gaps in the process of implementation, to support organisations, funders and researchers to learn from their experience and make improvements.^19 20^

The GIAF can be applied at different levels of impact assessment—a mini, standard or maxi impact assessment. Parry and Stevens describe the mini impact assessment as a ‘desk top exercise’, the standard with greater quantification of impacts and a maxi as an extensive collection of new data and quantification.^21^ The GIAF mini impact assessment involves rating a project across 1–3 GIAF domains (ladders), a standard involves use of checklists across four domains and a maxi impact assessment involves five or more domains.

While several domains and the relevant checklists of the GIAF have been used in previous projects within education settings, clinical guidelines, digital mental health and social and healthcare. The European Platform to Promote Well-being and Health in the Workplace (EMPOWER) is the first multicountry project that will involve a maxi impact analysis involving multiple domains to test the use of both the initiation and maturity phases of the GIAF model. This project is conducted by a consortium of 15 organisations across 9 countries (8 European countries and Australia). All consortium partners are listed in the author’s note at the end of this paper. This project aims to develop and implement an occupational e-Mental Health Intervention in the workplace involving a digital platform (webpage and app). The approach for effectiveness analysis is a cluster RCT in three countries (Finland, Poland and Spain). The project commenced in January 2020 and will finish in June 2024. The EMPOWER platform has been developed especially for small to medium-sized enterprises (SMEs) and public agencies. The UK joined the consortium as a fourth demonstration site but will not participate in the impact analysis. Further information is available in the study protocol of EMPOWER.^22^

Stakeholders and potential EMPOWER platform users (eg, employees, employers, work health and safety personnel) have been involved in the design and development phases of the EMPOWER platform.^22^ Stakeholders continue to be involved in other aspects of the research performed by different consortium partners involved in the EMPOWER platform, including identifying barriers and facilitators, and development of policy recommendations as part of this study.

There are four independent evaluations of the EMPOWER platform performed by different groups within the consortium. These are:

1. Evaluation of intervention effectiveness (outcomes for individuals who use the EMPOWER App). This study will be conducted by Parc Sanitari Sant Joan de Déu, Sant Boi de Llobregat, Spain.
2. A realist evaluation of the facilitators and barriers perceived by employers and employees on the use of the EMPOWER platform. This study will be conducted by Erasmus University Rotterdam, Rotterdam, the Netherlands.
3. A cost-effectiveness analysis of the EMPOWER platform. This study will be conducted by Erasmus University Rotterdam, Rotterdam, the Netherlands.
4. An impact analysis of the processes to develop and implement the EMPOWER platform. This study will be conducted by the University of Canberra, Canberra, Australia.

The topic of this manuscript is study 4, the impact analysis.

### Objective and aims

The aim of this protocol paper is to describe the rationale and methods that will be used to conduct an impact analysis of the EMPOWER project using the GIAF methodology. The objective of the manuscript is to present the GIAF methodology and how it will be used in the impact analysis of the EMPOWER platform in three European countries (Finland, Poland and Spain).

### Research questions

The research questions for the study are as follows:

1. To what extent can the GIAF Framework domains measure the development (Initiation) and early implementation (Maturity) phases of the EMPOWER platform in each country?
2. Can the GIAF Framework be used to conduct a comparative analysis of the implementation of the EMPOWER platform across three countries? To what extent is the GIAF framework useful for measuring implementation process domains in order to provide reliable and informative answers to these research questions?

## Method and analysis

### GIAF toolkit

The impact analysis of the EMPOWER platform in three countries will use qualitative and quantitative analysis using the novel GIAF methodology.^20^ The GIAF methodology involves a framework and toolkit to assess the three main phases of implementation research (Initiation, Early Implementation or Maturity and Late Implementation or Evolution).^20^ For the EMPOWER study, the first two phases of implementation will be evaluated. The relevant GIAF tools are described below.

#### GIAF glossary of terms

The GIAF glossary of terms was developed and refined through a consensus-based approach with the collaboration and validation by an international panel of experts in implementation research and health policy to generate a common language for communication across different disciplines, research areas and countries. Prior development of glossaries of terms used the same approach in health services and systems research^23^ and in the analysis of healthy habits and longevity.^24^ The GIAF glossary provides a standard definition of 180 terms relevant to implementation and impact analysis and the GIAF methodology. Excerpts of the GIAF glossary and the definitions of the terms relevant to this study are provided in online supplemental appendix 1. They include the first two phases of implementation (initiation and maturity), the three main domains of Initiation (planning, pre-engagement and prereadiness), and the five domains of maturity (readiness, usability, dissemination, adoption and uptake).

#### GIAF taxonomy

**Figure 1:**
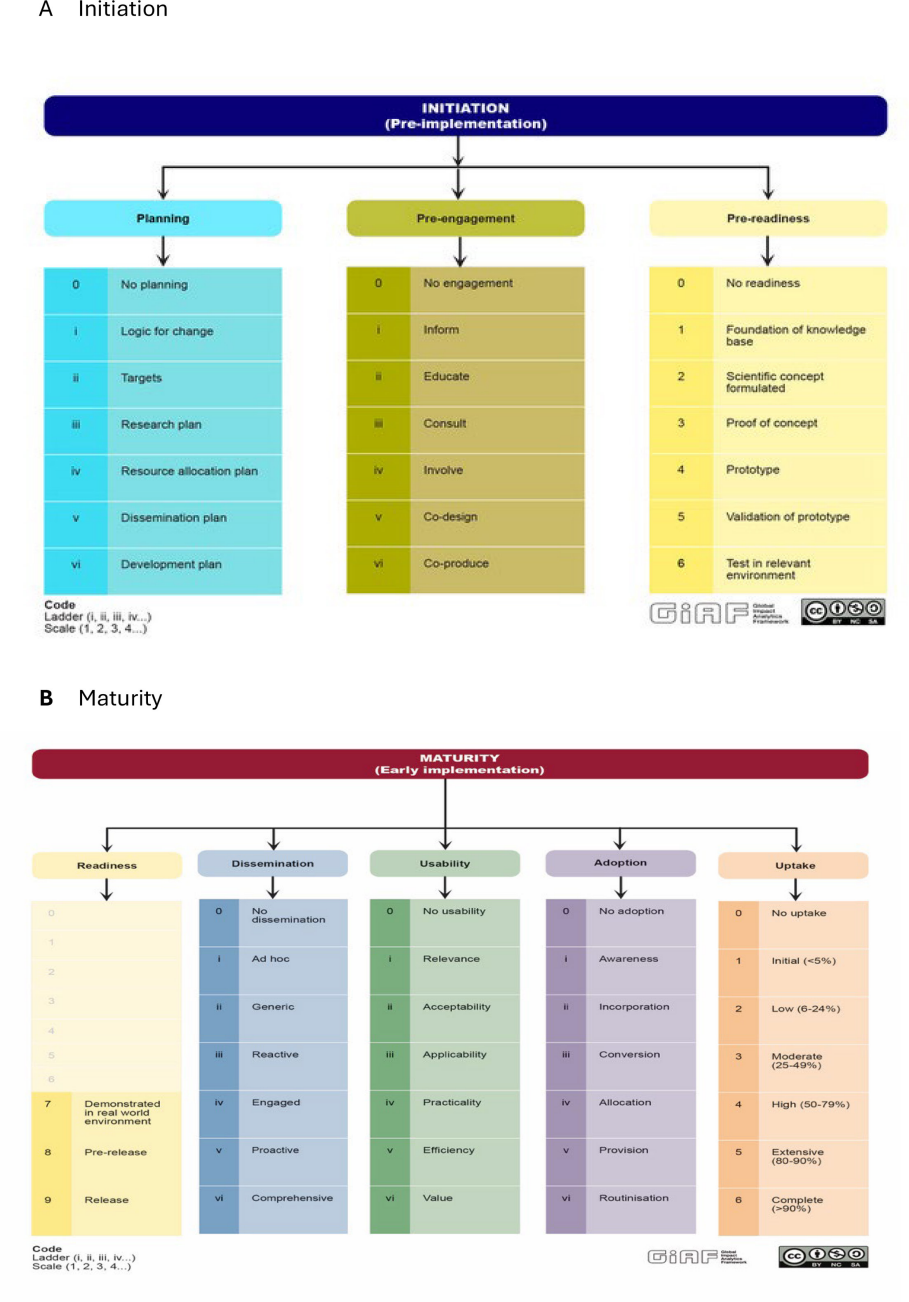
The two phases and domains of the GIAF impact analysis methodology for the EMPOWER platform (the process of Initiation and Maturity (Early implementation)). EMPOWER, European Platform to Promote Well-being and Health in the Workplace; GIAF, Global Impact Analytics Framework.

The hierarchical taxonomy of the process of impact analysis in implementation research is shown in figure 1. This is an ontology-based classification system that follows two main rules: <part of>which defines parent and child categories in the hierarchical tree and <is a> or operational and mutually exclusive definitions included in the glossary of terms. It represents the three main domains of Initiation and the five main domains of maturity as well as their 53 subdomains or child categories. For example, ‘adoption’ is divided into seven subdomains (no adoption, awareness, incorporation, conversion, allocation, provision and routinisation) (refer to the definitions in online supplemental appendix 1).

#### Measurement and the GIAF checklists

One of the challenges of impact analysis is how to harmonise measurement of different domains that are categorical, ordinal or numerical and that can be adapted to the conditions of highly heterogeneous programmes and projects, requiring different coding and rating approaches. To overcome these challenges, we have developed three rating and representation systems of the results of the GIAF checklists: scales, ladders and profiles. For example, in the case of maturity or early implementation, only the domain ‘uptake’ follows a defined ordinal ranking (figure 1 taxonomy). Readiness, dissemination and adoption follow a quasi-ordinal ranking. These categories are typically labelled in a way that suggests an ordinal progression, but the degree of difference between the categories cannot be clearly defined. In addition, a step represented by one item in the checklist can be skipped or merged with the next step in a particular study. We call this ranking approach a ‘ladder’ defined as a measurement of the process in implementation that provides sequential levels which assign a qualitative gradient/meaning to complex entities which are not scalable. The difference between the levels of a ladder is not necessarily of equal or sequential value and does not imply a gradual progression across levels. In the real world, a ladder level can be skipped in a specific context, whereas in a rating scale, this is not possible.

For every domain, regardless of whether it is a scale, ladder or profile, there is a recommended rating technique using a checklist. A checklist is a collection of the nominal items which are directly linked to each level of the domain. Most of the GIAF checklists are based on prior standardised instruments. The planning checklist is an adaptation of several planning templates.^16 25^ We have adapted the Technology Readiness Level version of the Horizon programme at the European Union for the prereadiness domain (onitiation phase) and readiness domain (maturity phase)^26^ and assessed its feasibility and inter-rater reliability in a separate study.^27^ The usability checklist has been validated in previous studies.^17 26 28^ The dissemination checklist was iteratively developed but based on the WHO International Classification of Health Interventions categories of target, action and means,^29^ and the uptake scale has been adapted from.^30^ Finally, the adoption impact ladder was developed by our group and validated.^17 31 32^ It is preferable that the rating for each domain is identified by more than one researcher (two or more raters), and there is agreement across the researchers and stakeholders in every study. Peer-reviewed publications on the GIAF toolkit, validation studies and its uses in the real world are ongoing.^17 27 28 33^ Refer to the GIAF University of Canberra website for further details.^20^ Each checklist is ~10 pages long and consists of a glossary of terms, instructions for the rater, and each level contains the characteristics of that level, a description and examples, and level completion criteria. Table 1 shows an example of level (i) of the GIAF Planning checklist.

**Table 1:** Level (i) of the GIAF Planning checklist

Level	Name	Characteristics	Description and examples	Level completion criteriaVerifiable information tick whether you deem the level is completed then document the information used to make your decision (documents, data, sources, opportunistic information). If there is not enough information to make a decision whether the criteria have been met for a level, please note this in the space provided.
i	Logic for change	Logic for change is a schematic representation which explains how the programme is intended to work, the process including the relationship between actions/activities and outcomes.Logic models are based on the understanding of how change happens.They are an explanatory account of how and why programmes give rise to outcomes.	A logic model is a tool used in programme planning and evaluation. It outlines a sequence of inputs (resources), activities (processes), outputs (results). It focuses on describing the logical flow of a programme without providing detailed explanations of the causal mechanisms.They can be created using different techniques and formats, such as flow charts, maps, tables, narrative or programme on a page. 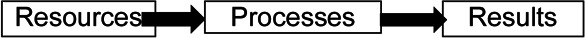	The logic for change is expressed in some form.Criteria Met? 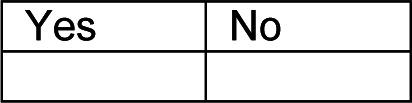
				Please document/comment below the information used to make your decision and note if there are any gaps in the logic model.

GIAF, Global Impact Analytics Framework.

### European Platform to Promote Well-being and Health in the Workplace

The EMPOWER app, available for use by employees, comprises four components:

- Screening for psychosocial working conditions (eg, symptomology, work functioning) and screening for physical health, mental health and absenteeism.
- Recommendations to reduce psychosocial stress at work.
- Promotion of workplace mental health and well-being for example, psychoeducation, tools to reduce stress, promotion of healthy lifestyles and increase well-being.
- Modules to reduce absenteeism and presenteeism including psychoeducation and problem-solving.

The screening tool, the Psychosocial Stressors at Work Scale (PSWS), is a 16-item measure of psychosocial working conditions such as work overload, conflicts at work, burn-out, bullying and harassment. The results of the PSWS are then matched with recommendations for employees based on their level of stress.

The EMPOWER website contains an employer portal which provides summarised feedback to employers on the mental health of employees and recommendations for employers to address employee work-related stress and mental health issues.

### Implementation sites and target audience

The EMPOWER platform will be implemented in small-medium enterprises and public companies from a variety of sectors in three cities and regions in Europe: Barcelona (Catalonia, Spain), Turku (Finland), Lodz (Poland). Three European countries were selected to represent different European welfare and health service models: Finland (represents the Scandinavian model), Spain (Mediterranean model), Poland (Eastern European model). Two target audiences will be considered: (1) the employees from the participating companies (primary target of the EMPOWER project) and (2) the employers of these companies (secondary target).

All employees from the selected companies meeting the criteria will be invited to participate in the cluster RCT. The inclusion criteria for participants are: (1) being aged 18 years or older; (2) having a mobile phone with Internet access; (3) having sufficient knowledge of the local language and (4) giving informed consent. Once these criteria are met, participants will be invited to download and use the app. After obtaining informed consent, employees will be asked to complete several questionnaires and will then be guided through different modules and content sections of the EMPOWER app.^22^ All employee participants will answer a total of three assessments via survey through the EMPOWER app. This allows for separate analyses at the country and company levels and the level of the EMPOWER cluster RCT.

Companies will be selected based on being a SME or public agency (<250 employees). The country coordinators will approach companies who meet the inclusion criteria, provide information about the study and the EMPOWER platform and invite them to participate. Those who consent will enter into a contract between the researchers and employers to affirm ethical considerations, data collection and communications. Each workplace (and thus the employees) will be randomised into the RCT.

### Unit of analysis

The units of analysis are the key domains of the process of implementation throughout the two phases (Initiation, Maturity) (figure 1). In the Initiation phase, analysis will be at project level only (across the three countries). In the maturity phase, there will be an analysis of the impact of the implementation of the EMPOWER project, subgroup analysis at the country level (Finland, Poland and Spain) and some domains at the level of the recruited organisation/workplace.

### Training and preparation for data collection

The preparation for the data collection commenced prior to finalisation of the EMPOWER app development and commencement of the RCT. The country coordinators are researchers from universities in each country and members of the EMPOWER consortium. They are located in each country (Finland, Poland and Spain) and have attended training. Their tasks include monitoring and managing the trial fieldwork in their country, data entry and validation, solving problems during the fieldwork and will remain in constant communication with other EMPOWER consortium partners. For the impact analysis data collection, there were three orientation/training sessions with the country coordinators. In October 2021, an introduction to the impact analysis and explanation of the GIAF framework was conducted online for country coordinators and included an orientation on the dissemination run sheet (a spreadsheet with predetermined categories), used to collect data for the dissemination domain (maturity phase). The run-sheet enables concurrent collection (rather than retrospectively) of the parameters of dissemination activities (eg, the communication frequency with an employer). Country coordinators enter data on the activities to recruit organisations/workplaces and to make the EMPOWER platform known and make it used. An additional training session was held in April 2022 with regards to the dissemination run sheet, and a third meeting in September 2022 on the usability and adoption questionnaires to be used at the end of the recruitment period. The usability and adoption questionnaires (for employers) are conducted through a semistructured interview with the employer representatives. In preparation for the data collection on the usability, the questionnaire was first refined to the three country contexts in English. Each country coordinator translated the questions into their country language (all being fluent speakers of the language). There was subsequent collaboration with each of the country coordinators to culturally adapt the questions, yet ensure they remained within the integrity of the intent of the questions. Finally, each country coordinator translated the usability questions into the relevant language. Support to understand and use the GIAF tools to collect information relevant for the dissemination, usability and adoption domains will be provided to country coordinators as needed.

### Data collection methods

The GIAF methodology uses an approach involving checklists and which use diverse methods of data collection and from multiple sources including end users (employees of companies), employers, country coordinators and other consortium groups (eg, development and implementation groups) involved in reporting development progress and dissemination plans and activities. For each domain in the initiation and maturity phase, the data collection will be via a combination of the following sources of information (table 2).

**Table 2:** GIAF data collection methods and sources

GIAF phase	Domains	Data collection
Initiation	PlanningPre-engagementPrereadiness	EMPOWER consortium documents (project proposal, consortium reports) with descriptors to extract specific information.
		Records of opportunistic information known to the Impact analysis researchers through working with EMPOWER consortium partners and country coordinators throughout the project, via direct observation, face-to-face, phone or email discussions or purposeful approaches to individuals, for example, country coordinators.
		Project progress reports, deliverable documents, consortium publications, meeting minutes and other documents with respect to development of the EMPOWER project and platform testing, prior to implementation
Maturity	Usability	End users—standardised surveys from the GIAF toolkit to collect information.
	Dissemination	Country coordinators—The GIAF toolkit dissemination spreadsheet with predetermined dissemination activity categories and definitions for each country coordinator to concurrently record dissemination activities.
	UsabilityAdoption	Employer representatives—Standardised questions by the country coordinators via a semi-structured interviews and/or survey with key workplace personnel.
	Uptake	EMPOWER website and app usage data from users (employees and employers as a secondary target audience).
Other	Contextual information–barriers and facilitators	Country coordinators—an interview on completion of the recruitment for the EMPOWER app regarding country-level barriers and facilitators.

EMPOWER, European Platform to Promote Well-being and Health in the Workplace; GIAF, Global Impact Analytics Framework.

The users (employees) of the app will be asked questions for the usability domain (maturity phase) via the survey platform Qualtrics. The three country translations of the usability questions will be uploaded to Qualtrics for distribution via email using a dedicated link to each user in the workplace. The questions will be sent to employees at 6–8 weeks after the completion of their 7-week use of the EMPOWER intervention. If there is no response after 3 weeks, a reminder will be sent at 4 weeks. Each usability question will be rated on a 7-point Likert scale ranging from 1=strongly disagree to 7=strongly agree. After each question, there will be a space for a free text comment.

The employers/their representatives will be invited to answer the usability domain questionnaire via an interview with the country coordinators up to 2 months after recruitment and the EMPOWER app use has ceased at that workplace. The timing depends on the progress of recruitment at the time. There will be slightly different wording for the usability questions to the employer, because of their context and level of knowledge (eg, budget, available resources). Employer responses will be rated on the same 7-point Likert scale (strongly disagree to strongly agree).

As the bilingual country coordinators are part of the EMPOWER consortium, they will ask the employers the adoption domain questions at the same time as the usability questions. Answers will be recorded on the paper-based questionnaire as free text. Survey and interview responses and dissemination spreadsheets were written by the country coordinators in English which was then provided to the University of Canberra impact analysis team.

The name of the workplace (not the representative’s name) and position of the responder in the company (eg, employer, employed manager, supervisor, work health and safety representative) will be made known to the University of Canberra team. The name of the workplace is necessary so the number of employees in each workplace is known, and uptake (the uptake by employees) of the intervention (EMPOWER platform) can be calculated.

### Organisational learnings

Organisational learnings (formative and summative) are a process in which an organisation’s members actively use the knowledge acquired during the project to guide behaviour in such a way to promote the ongoing adaptation and improvement of the organisation.^34^,^36^ In this context, organisational learning refers to learnings for the EMPOWER consortium about the EMPOWER intervention and its development and implementation, arising from the Impact Analysis. We will use all information gained from each of the initiation and maturity domains from each country and synthesise it to generate new insights across the three countries which have the potential to inform and improve future mental health e-health interventions and implementation processes at the workplace. We aim to describe the impacts of the EMPOWER project and provide insights into what worked, in what context and why.

Formative organisational learning will be collected throughout the initiation phase and the preparation of annual reports, related meetings, reports and general communications. The summative organisational learnings will be validated through two processes. First, the targeted feedback on country-level barriers and facilitators that will be collected by University of Canberra from each country team (Finland, Poland and Spain) via a semistructured interview. Existing implementation frameworks have been used to develop the focus questions for the semistructured interview.^11 16 37 38^ Second, two expert panels (consortium partners and country representatives) will generate the summative organisational learnings and validate them through consensus in conjunction with other consortium partners. For example, employee and employer feedback that will be obtained through the realist evaluation performed by the Erasmus team; the information from the stakeholder survey conducted by the Fondazione IRCCS Istiututo Neurologico Carlo Besta (paper under review); stakeholder focus groups and scoping review,^10^ and the results of the impact analysis will be used to compare and affirm key organisational learnings from all consortium partners and stakeholders.

### Analysis

The approach to the analysis translates the narrative and the qualitative information to quantitative data within the GIAF framework for further analysis. The analysis will use mixed methods from the qualitative data gathered to rate on standardised ladders, profiles and scales in the GIAF toolkit. Four researchers from the University of Canberra team will rate the project independently on the various domains.

A case study of the EMPOWER platform intervention will be developed, containing all information necessary for raters to rate each GIAF domain and will contain references to the relevant information sources. Each rater will document on each checklist, the evidence (derived from the data collected relevant to that domain) for their determination of the rating level. Means will be calculated from employee and employer usability scores and mapped to the usability checklist and scored. Uptake will be calculated based on the number of employees at each company/country divided by the number of EMPOWER users as a percent and mapped onto the checklist and scored. Adoption responses will be mapped onto the adoption checklist and scored. Dissemination will be rated using the relevant checklist and given a score using the ladder. Engagement will be scored based on information from the various meeting notes and reports.

If differences occur between raters in the level on the ladder/scale/profile, the ratings will be discussed among the University of Canberra team until a consensus is reached for the final rating of the project on each domain. The degree of agreement across raters will be provided in addition to the final ratings.

Statistical analysis will include descriptive statistics (mean, percent) as well as the analysis of the relationships between domains (variables) and the comparison of the usability of the tool evaluated by the various participant groups after the completion of the RCT (randomised controlled trial). The analysis will include non-parametric tests (correlations and median comparisons) where relevant. Both qualitative and quantitative approaches will also inform key learnings. For example, quantitative data from the usability questions will be compared with qualitative adoption responses and quantitative uptake calculations. This will provide important information such as the extent to which employees in each country considered EMPOWER useful, relevant and of value. This measurement of usefulness may positively influence the degree that EMPOWER is adopted and the extent of the uptake into the target audiences. Country level barriers and facilitators (from country coordinator interviews and information from the realist evaluation (employee and employer feedback) will provide context for high or low ratings.

#### Participant and public involvement

Stakeholders and potential EMPOWER platform users (eg, employees, employers, work health and safety personnel) have been involved in the design and development phases of the EMPOWER platform.^22^ Stakeholders continue to be involved in other aspects of the research performed by different consortium partners involved in the EMPOWER platform, including identifying barriers and facilitators, development of policy recommendations as part of this study (refer to section on analysis).

## Ethics and dissemination

The impact analysis study protocol has been approved by the Research Ethics Committees of the University of Canberra (ID:202311841) and also the Fundació Sant Joan de Déu (PIC-39-20). The participating countries for the RCT (EMPOWER study) also obtained ethical approval through their respective ethical organisations in the participating countries. The impact analysis is registered with the Open Science Framework ID osf.io/eysc9. The EMPOWER project trial is registered at ClinicalTrial.gov with trial ID NCT04907604. The outcomes of the impact analysis study will be disseminated via conference presentations, peer-reviewed journals and key organisational learnings presented in relevant forums.

## Discussion

There are complexities with impact analysis of implementation studies such as the EMPOWER project with multiple components and the interactions to consider. Some examples are societal events (eg, labour-related employee strikes at the workplace); national events (eg, the advent of war in a neighbouring country); the timing and impacts of the COVID-19 pandemic on workplaces and employees; cultural factors (eg, stigma associated with mental health); capacity and skill (eg, employers’ communication approach to recruitment of employees to use the EMPOWER app). Data source and measurement (who, what, how and when) is critical in implementation research. The ontoterminology approach used in the development of the GIAF methodology, as demonstrated in health services research,^39^ assists in the analysis of some of this complexity by providing a toolkit. The GIAF methodology provides consistency with terminology, reduces ambiguity and clearly identifies phases and domains of the process (throughputs) of implementation research and how they relate to each other.

The EMPOWER project impact analysis will evaluate the process of the development and implementation; the processes involved in its development and ‘how’ and ‘to what extent’ the EMPOWER intervention was implemented in three countries (Finland, Poland and Spain), and the impacts on both primary and secondary target audiences. The overall EMPOWER project’s impacts from the development and implementation across the three countries will also be evaluated.

The information gained through the impact analysis will be relevant to the funder of EMPOWER (EU Commission), the primary target population (employees working mainly in small and medium-sized companies in Europe), and for organisational learning for all participating centres and workplaces involved. The impact analysis also aims to provide information to the EMPOWER consortium for the development of EMPOWER policy recommendations, future steps and for the potential spread of the EMPOWER intervention after the study. The results will be disseminated through publications and conferences and will potentially inform the implementation of similar platforms in other real-world contexts. Finally, there will also be learnings for future impact analysis and the GIAF methodology.

### General statement

No specific reporting guideline for impact analysis of implementation research protocols was identified via a search of the EQUATOR Network repository.^40^ The ‘Reporting guidelines relevant to implementation studies’ identified 26 relevant guidelines.^15^ After reviewing the 26 guidelines, the paper authors pragmatically used a combination of guidance from the Standards for reporting implementation studies guidelines (2017), the Six practical recommendations developed by Lengnick-Hall *et al*^15^ and the iCHECK-DH guidelines for reporting on digital health implementations (2023) to develop this paper structure.^15 41 42^

## Supplementary material

10.1136/bmjopen-2023-082219 online supplemental file 1
