# Supplementary material for: A protocol using mixed methods for the impact analysis of the implementation of the EMPOWER project: an eHealth intervention to promote mental health and well-being in European workplaces
Source: BMJ Open. 2025 Apr 9;15(4):e082219. doi: 10.1136/bmjopen-2023-082219 (PMC11987133; doi:10.1136/bmjopen-2023-082219)
Supplement: online supplemental file 1 [file bmjopen-15-4-s001.docx]

**Appendix 1 Excerpts from the GIAF Glossary – definition of terms relevant for the EMPOWER impact analysis protocol.**

1. Adoption: The level to which a target user or organisation takes the application of the emerging scientific knowledge as its own.
2. Allocation: The action taken by the target user/organisation to grant resources for the implementation of the application of the emerging scientific knowledge.
3. Application: Any output of the emerging scientific knowledge that can be implemented in the real-world (e.g., policy, program, service, intervention, product or technology). Adapted from [1].
4. Assimilation: The actions that indicate the nominated representative of a target organisation, accept, and can use the application of the emerging scientific knowledge as part of their own knowledge base. It can include mentions initiated by the target organisation in the media.
5. Awareness: The actions that indicate that defined individuals representing the target audience are cognizant of the emerging scientific knowledge or its application. Evidence should be provided, that there is action taken to improve knowledge on the topic and provided feedback.
6. Conversion: The actions taken for translating the emerging scientific knowledge and its application from the individuals who have adopted it for application in the target organisation. These actions may include legislation, plans, policy programs, regulatory norms, rules, and official or corporate indicators).
7. Dissemination: The act of making the emerging scientific knowledge or its application ‘known and making it used” to the target audience so that it reaches, is made known to or available to facilitate the uptake and use.
8. Emerging scientific knowledge: Knowledge that has successfully passed the phases of discovery and corroboration and has an application ready for implementation in the real-world setting.
9. Engagement: Is a two directional interaction and relationship between the sender and recipient of the emerging scientific knowledge and its application. It should increase the understanding of the new knowledge by the recipient and advance its implementation in the real world. Adapted from [2, 3].
10. Impact analysis: The evaluation of the effects of an application of emerging scientific knowledge on a targeted audience in a specific real-world context. This includes the direct and indirect, intended, and unintended, and positive or negative results.
11. Implementation: The planning, process, and actioning of the application of the emerging scientific knowledge in the real-world.
12. Initiation: The phase of starting an implementation project (pre-implementation) that includes the study of its planning, pre-engagement and the pre-readiness of the prototype.
13. Ladder: A measurement of the process in implementation and complexity research. It provides sequential levels which assign a qualitative gradient/meaning to complex entities which are not scalable. The difference between the levels of a ladder are not necessarily of equal or sequential value, and do not imply a gradual progression across levels. In the real world a ladder level can be skipped in a specific context, whereas in a rating scale this is not possible.
14. Maturity: The phase in which the application of the emerging scientific knowledge is tested in the real world for the first time or in a new context (early implementation), and includes the study of its readiness, usability, dissemination, adoption, and uptake.
15. Uptake: The level of adoption of the application of the emerging scientific knowledge in a target audience at the end of the maturity phase.
16. Planning: Is the process of thinking and formalising in a written document, the steps and activities required to achieve an explicit goal. It includes deciding on the tasks in advance to achieve the implementation of a project; the what, when, where, why and how and who will do the activities.
17. Pre-readiness: The level of preparedness of an application of the emerging scientific knowledge, where the knowledge base is established, a scientific concept is formulated, there is proof of the concept, a protype is developed, validated, and tested in a relevant environment.
18. Provision: All the supply system available for producing results of using the emerging scientific knowledge e.g., staff, services, interventions, and technologies.
19. Readiness: The level of preparedness of an application of the emerging scientific knowledge to be used in the real-world and for its release, marketing, commercialisation, or open access.
20. Routinisation: The target individual or organisation has incorporated the application of the emerging scientific knowledge into its regular procedures.
21. Scale: Is an ordinal sequential value which follows gradual progression without missing any level. e.g. 0<1<2<3.
22. Target audience: Is the number and representativeness of individuals/organisations identified and selected as potential users of the application of the emerging scientific knowledge. Typically, the target audience is defined within a specific context- geographical or other (e.g., a health district, a country or health system). The study sample is a sub-set of the target audience using a pre-defined selection method within an implementation project. (new)There are:
    1. Target user: are the individual/s
    2. Target population: the set of individuals
    3. Target organisation: are companies, institutions, or associations with a particular purpose. The organisation is typically represented by nominated individuals.
23. Usability: A measure of the usefulness of the application of the emerging scientific knowledge during the maturity phase. The main domains of usability relevance, acceptability, applicability, practicality, efficiency, value.
24. Usefulness: The practical advantages of using the emerging scientific knowledge or its application throughout any phase of implementation.

References:

1. Goodrich DE, et al., *Quality Enhancement Research Initiative. QUERI Roadmap for Implementation and Quality Improvement*, United States Department of Veterans Affairs Veterans Health Administration Office of Research and Development Health Services Research and Development, Editor. 2020, U.S.A.Department of Veterans Affairs,: U.S.A.

2. Brodie, R.J., et al., *Consumer engagement in a virtual brand community: An exploratory analysis.* Journal of Business Research, 2013. **66**(1): p. 105-114.

3. Hollebeek, L.D., et al., *Customer engagement in evolving technological environments: Synopsis and guiding propositions.* European Journal of Marketing, 2019.
