# Supplementary material for: A protocol using mixed methods for the impact analysis of the implementation of the EMPOWER project: an eHealth intervention to promote mental health and well-being in European workplaces
Source: BMJ Open. 2025 Apr 9;15(4):e082219. doi: 10.1136/bmjopen-2023-082219 (PMC11987133; doi:10.1136/bmjopen-2023-082219)
Supplement: Supplementary file 2 [file bmjopen-2023-082219.reviewer_comments.pdf]

PEER REVIEW HISTORY

BMJ Open publishes all reviews undertaken for accepted manuscripts. Reviewers are asked to complete a checklist review form and are provided with free text boxes to elaborate on their assessment. These free text comments are reproduced below.

ARTICLE DETAILS

Title (Provisional)

Study Protocol for the Impact Analysis of the EMPOWER project: an eHealth intervention to promote mental health and wellbeing in European workplaces

Authors

Salvador-Carulla, Luis; Lukersmith, Sue; Woods, Cindy E; Chen, Tom; de Miquel, Carlota

---

VERSION 1 - REVIEW

**Reviewer**	**1**
**Name**	**Aust, Birgit**
**Affiliation**	**Det Nationale Forskningscenter for Arbejdsmiljø**
**Date**	**12-Feb-2024**

COI **As I pointed out before agreeing to review this manuscript, I was myself part of a EU-Horizon financed research project which is another project that was started by the same EU-call on mental health at work. Our project with the name MENTUPP has been conducted during the last four years and we are currently writing up the final results from our research project, including effect and process evaluation results. Members of both research groups (EMPOWER and MENTUPP) have met (e.g. conducted a symposium together about their respective research projects), however I was not personally a part of that. I do not see any conflict of interest in reviewing this manuscript about a study protocol for the impact analysis of the EMPOWER project, but made aware of this before I started reviewing the manuscript. BMJ Open Managing Editor, Ms. Shona Reeves, asked me to mention that we discussed this issue in this section.**

This is an interesting manuscript presenting the study protocol for the impact analysis of the EMPOWER project, an eHealth intervention to promote mental health and wellbeing in European workplaces. The authors argue for a better and more standardized evaluation of what actually happens during the implementation of workplace interventions and explain in detail how they are planning to assess this by focusing on the initiation and the early implementation phase. The manuscript gives a lot of information about the main approach

that will be used for this assessment (the GIAF toolkit), which certainly is an interesting contribution to implementation research. However, other methods for data collection and analysis that also will be used, are only very broadly described. In addition, some more information about the intervention are needed to better understand what it is that will be assessed and analyzed.

Here are my suggestions for further improvement of the manuscript:

General remarks:

I have a general remark about the term “impact analysis”. For me the term “impact” implies more something that refers to the effects and consequences of an intervention, for example to what degree the EMPOWER intervention led to less absenteeism, therefore less costs in the health care system or other types of changes, for example healthier workplaces. However, it seems the authors use “impact” analysis in a way I would use “implementation analysis”, which I would define as the way the intervention was actually conducted (was it used, adopted etc.). The authors seem to not distinguish between “impact analysis” and “implementation analysis” but seem to mean the same with both terms. However, in the provided GIAF glossary separate definitions for “Impact analysis” (No. 10) and “Implementation” (No. 11) are given which refer more to effect (impact) and application (implementation). Maybe the confusion stems from the fact that only two of the three phases of the Global Impact Analytical Framework (GIAF) are used for the analysis presented here. It seems that the first two phases of the GIAF relate more to implementation, while the third phase (Evolution phase) might relate more to impact – but I might be wrong. However, it would be good to clarify these terms and to explain why the authors chose to call their analysis “impact analysis” instead of “implementation analysis”.

However, - to not make things more complicated than necessary - in the following comments I will use the term “impact analysis” just like the authors did in the manuscript.

Comments to the different parts of the manuscript

Abstract:

In the introduction section in the abstract where the authors describe the aims of this study, it should be made more clear that the aim of this protocol paper is to present the rational and the methods that will be used in order to conduct an impact analysis, rather than the aims of the impact analysis.

Also, some sentences in “Method and analysis” section in the abstract sound more like a reporting of what already has been done rather than what is planned to be done. Please check and change.

Strengths and limitations

The bullet points about strengths and limitations should be focusing more on the strengths and limitations of the methods presented in this protocol paper, rather than the contribution

provided by the results that are expected from conducting the impact analysis. In addition, potential or actual limitations of this approach should also be mentioned. For example, it seems to be that the GIAF toolkit, which is the central method presented here, has mostly been used in health service research (as mentioned by the authors in the discussion, page 10, line 22) and therefore it remains to be shown if it also can be used to evaluate the EMPOWER intervention which is conducted in workplaces.

Introduction

Page 4, line 18: Please be more specific with regard to describing the topic of this manuscript. It is the presentation of the methods used for the impact analysis – not the impact analysis itself.

Page 4 Objective and aims

Also, in this part it seems like the objective and aims of the impact analysis are too much in focus, rather than the aim of a protocol paper explaining how this will be done (the methods). Instead of highlighting the research questions for the impact analysis, I suggest to highlight more the aims of the protocol paper, which are to show how these aspects can be measured in order to be able to give reliable and informative answers these research questions.

Page 6, line 51: The authors write “The GIAF defines profile as a visual representation that synthesises the information related to relevant domains of a construct or entity. Typically, a profile represents a series of sub-domains of a main entity that cannot be arranged in an ordinal scale or aggregated.” Would it be possible to give a visual example of such a profile? The description remains very abstract and it would be good to see an example of such a visual representation of a profile.

Page 6-7, the paragraph about the GIAF checklists: It would be very informative to show an example of such a checklist to get a better understanding for what actually will be measured and assessed.

Page 7, section about “Implementation sites and target audience”

I do not understand what is meant with “Three target audiences will be considered” – considered for what? The intervention? And why is the Empower consortium (who developed the intervention and will evaluate it) also a target audience for the intervention? Please explain.

In the second paragraph in this section the inclusion criteria for the individual employees are explained in detail. However, no information about the inclusion criteria for the workplaces is given and also no information about what kind of information are assessed from the employers (secondary target for the intervention). Please also add some information about, what the intervention actually consists of. Otherwise, it remains very abstract what employees and employers/workplaces should be ready for, what they should use, disseminate and adopt. By adding some information about these aspects it will be possible

to describe how the measurements will be applied to the EMPOWER intervention. Moreover, please add some information about the type of workplaces participating in the EMPOWER intervention. Do they come from certain sectors?

Page 7, section about “Training and preparation for data collection”

Please explain who the Country Coordinators and the Country Coordinator teams are. Are they part of the research team?

Page 8, section about “Data collection methods”

The authors write: “The GIAF methodology uses an approach involving the checklists and which use diverse methods of data collection and from multiple sources including end users, employers, Country Coordinators and other consortium groups involved in reporting and dissemination. For each domain in the Initiation and Maturity phase, the data collection is via a combination of the following sources of information (the Phase and domain where this method is used is in brackets):”

Please explain: Who are the “end users”? What “other consortium groups” are meant here? What is their role and what kind of data do they provide?

The list that follows this paragraph is difficult to understand. Could these information be given in a more informative table where it becomes more clear who is asked about what – referring to the domains from the GIAF framework that were described in detail before? As the list is now, it is difficult to see how this relates to the data collection with regard to the GIAF toolkit.

Page 9, line 7: The authors write “There is slightly different wording for the usability questions to the employer, because of their perspective (e.g., budget, available resources).” Does that mean that you adjust the questionnaire to the employers according to their perspective? Please explain.

Page 9, line 8. The authors write: “The name of the workplace (not the representative’s name) and position of the responder in the company (e.g., employer, employed manager, supervisor, work health and safety etc.) will be made known to the UC team.” Please add why you need to know the name of the workplace.

(Also, it should be “work health and safety representative”.)

Page 9 section about “Participant and Public Involvement Statement”

I do not understand the purpose of this statement at this point of the manuscript. This seems to be something that should have been said much earlier when introducing the study and how the intervention was developed. Please move or integrate better.

Page 9, Section about Organizational learning

Considering that organizational learning is the topic of two of the three aims of the study presented on page 4 under Objective and aims, this section is very short and not very

precise compared to the many pages that were used to describe the assessment using the GIAF toolkit. The methods used for assessing organizational learning are described on a very broad level with references to many data sources that have not been introduced before.

In addition, it is not clear to me what actually is meant by "organizational learning". It seems not to be the learning that might take place in the participating workplaces/organizations, but rather the learning in the EMPOWER consortium about the EMPOWER intervention (see for example page 9, line 29-30: "We aim to describe the impacts of the EMPOWER project, and provide insights into what worked, in what context and why.")

The methods used for the analysis of organizational learning are also not mentioned in the abstract. Please explain what role the measurement of organizational learning plays in the impact analysis and adjust the level of detail in describing the methods to assess organizational learning accordingly.

Page 9, section about Analysis

Similarly, this section is rather short and only very broadly describes how the collected data will be analyzed. For example, I assume there is also a need to translate the answers from Finland, Poland and Spain into English before the four raters from the UC team can rate them for the various domains. Does the last sentence of this section "Both qualitative and quantitative approaches will also highlight key learnings." refer to the analysis of the data collected to assess organizational learnings? Please provide a more detailed description of how the variety of data that was mentioned in the section 2.8 about organizational learnings will be analyzed.

Page 10, Discussion

The authors write (line 27) "The EMPOWER project impact analysis will evaluate the process of the implementation; 'how' the EMPOWER intervention was implemented in three countries (Finland, Poland, Spain)"

I think it could be added "and to what extend" (after 'how')

The sentence continues with : ", and the impacts on both primary and secondary target audiences. The overall EMPOWER project's impacts will also be evaluated." Please explain what is meant by the "overall impact".

Minor comments

There are inconsistencies with regard to the numbering of sections: First sections are numbered, but then there is a gap between 2.1 and 2.8 (numbers are missing) and after the section 2.9 the sections are no longer numbered. While this might be less important for the introduction and discussion part, the method section (nr. 2) is very long and would gain from a structure that clearly distinguishes between main sections and relating subsections.

Page 6, line 38: Please check the sentence: "Readiness, dissemination and adoption, dissemination follow..." The "and" seems to be misplaced.

Page 6, line 51: “The GIAF defines profile as…” (should be profiles)

Page 8, line 12 “The Country Coordinators will also ask the employers the adoption domain questions at the same as the usability questions.” The word “time” seems to be missing after “same”

Page 9, line 57: “If differences occur between raters in the level on the ladder/scale/profile, the ratings are discussed amongst the UC team, and a consensus reached for the final rating of the project on each domain.” I assume you meant to write “until a consensus is reached”.

Page 10, line 11 : “There are complexities with impact analysis of implementation studies such as EMPOWER project with multiple components and the interactions to consider.” Add “the” before EMPOWER project.

**Reviewer**	**2**
**Name**	**Abbas, Ansar**
**Affiliation**	
**Date**	**09-Apr-2024**
**COI**	**no competing interest to be declared**

Good luck

**Reviewer**	**3**
**Name**	**Sun, Sijin**
**Affiliation**	**Imperial College London, Dyson School of Design**
**Engineering**	
**Date**	**13-Sep-2024**
**COI**	**No**

It is an interesting project, but I have some particular questions towards the methodologies of this study that might require clarifications:

1. It is mentioned EMPOWER peoject was "conducted by a consortium of 15 organisations across nine countries", eight of them are european, but only three countries were included in this study. Was there any particular reason?

2. For the target audiences, why were "members of the EMPOWER consortium including the local country coordinators" included? They obviously have different knowledgement towards this project and should not be compared with other participants. Or were they included here because they were interviewed with other particular questions?

3. It was stated that the local country coordinators translated the questions, will they also translate the qualitative results? Are there any bilingual researcher in the UC team? Could there be any cultural factors that may caused bias?

4. It was mentioned "The usability and adoption questionnaires (for both employees and employers) are conducted through a semi-structured interview with the employer representatives.". Who are the employer representatives? Throughout the study, could there be any pressure from the employer that might bias the results of the employee participants?

VERSION 1 - AUTHOR RESPONSE

**Reviewer Comment**	**Response**
Reviewer 1	
The manuscript gives a lot of information about the main approach that will be used for this assessment (the GIAF toolkit), which certainly is an interesting contribution to implementation research. However, other methods for data collection and analysis that also will be used, are only very broadly described.	The EMPOWER platform will undergo four evaluation studies, the effectiveness (of the intervention on individual outcomes e.g. depression and anxiety, reduction in presenteeism), a realist evaluation (on employees and employers feedback), cost effectiveness of the EMPOWER platform and the 4th is the impact analysis of the processes taken from the development phase of the intervention through to those processes undertaken to implement in European workplaces.<br><br>The methods and results for the first 3 studies will be reported in separate publications.<br>This has been clarified in the introduction.<br><br>The topic of this paper is the 4th study – the evaluation of the processes to develop and then implement the EMPOWER platform.<br><br>The text has been revised in the abstract and introduction to clarify.
In addition, some more information about the intervention are needed to better understand what it is that will be assessed and analyzed.	Information about the EMPOWER platform has been added in section 2.2
I have a general remark about the term "impact analysis". For me the term "impact" implies more something that refers to the effects and consequences of an intervention, for example to what degree the EMPOWER intervention led to less absenteeism, therefore	We thank the reviewer for this comment. We agree that impact refers to impacts on and consequences of the intervention. As per the definition of impact analysis it is the effects of the EMPOWER app on a specific target audience. For the EMPOWER app there are two target audiences, the individual employees and

less costs in the health care system or other types of changes, for example healthier workplaces. However, it seems the authors use "impact" analysis in a way I would use "implementation analysis", which I would define as the way the intervention was actually conducted (was it used, adopted etc.). The authors seem to not distinguish between "impact analysis" and "implementation analysis" but seem to mean the same with both terms. However, in the provided GIAF glossary separate definitions for "Impact analysis" (No. 10) and "Implementation" (No. 11) are given which refer more to effect (impact) and application (implementation). Maybe the confusion stems from the fact that only two of the three phases of the Global Impact Analytical Framework (GIAF) are used for the analysis presented here. It seems that the first two phases of the GIAF relate more to implementation, while the third phase (Evolution phase) might relate more to impact – but I might be wrong. However, it would be good to clarify these terms and to explain why the authors chose to call their analysis "impact analysis" instead of "implementation analysis".	the workplaces/employers. There are impacts for both. For individuals/employees it is the effects and consequences for themselves of using EMPOWER. For this EMPOWER platform it is also the effects of the implementation on the employers and the workplaces.<br><br>In this study the Impact analysis focuses on the processes and their impacts across **two phases** of implementation research (development and then implementation in the real world).<br>1. The analysis of the **development (Initiation) phase** of the EMPOWER Platform (the analysis of how and what was done to develop the intervention, and then get it 'ready' to be implemented in the real workplaces). Impact analysis of this component of real world research is critical – to identify the extent that end users were involved (which potentially impacts effectiveness of the intervention) and whether it has been adequately tested before being implemented to remove technical issues etc.<br>2. In the **implementation (Maturity) phase**, it focuses on the impacts of the processes of implementing it in the real world (the workplaces in the three countries) e.g. how it was made known in the workplaces, and how the researchers or workplace made it known to employees/encouraged their employees to use it, was it embedded in the workplace routine, whether employers and employees thought it was useful etc.<br><br>We analyse the development and implementation processes in this impact analysis.<br><br>These are the domains of impact that the GIAF measures. The GIAF measures how it was developed, how it was implemented and the impact of these processes. The third phase in GIAF measures its Evolution and spread (not relevant in this study).

Abstract:<br>In the introduction section in the abstract where the authors describe the aims of this study, it should be made more clear that the aim of this protocol paper is to present the rational and the methods that will be used in order to conduct an impact analysis, rather than the aims of the impact analysis.	The aim of the study has been changed to: The aim of this protocol paper is to describe the rationale and methods that will be used to conduct an impact analysis of the processes undertaken to develop and implement the EMPOWER project using the GIAF methodology.
Also, some sentences in "Method and analysis" section in the abstract sound more like a reporting of what already has been done rather than what is planned to be done. Please check and change.	We have proofread the Method and Analysis section in the abstract and body of the paper and have corrected the tense where necessary.
Strengths and limitations<br>The bullet points about strengths and limitations should be focusing more on the strengths and limitations of the methods presented in this protocol paper, rather than the contribution provided by the results that are expected from conducting the impact analysis.	Four additional dot points have been added to the strengths and limitations which focus on the methods presented rather than the expected results. Two of the original dot points have been deleted.
In addition, potential or actual limitations of this approach should also be mentioned. For example, it seems to be that the GIAF toolkit, which is the central method presented here, has mostly been used in health service research (as mentioned by the authors in the discussion, page 10, line 22) and therefore it remains to be shown if it also can be used to evaluate the EMPOWER intervention which is conducted in workplaces.	The text has been revised in the introduction to be: Whilst a number of domains and the checklists of the GIAF have been used in previous projects within education settings, the development of guidelines, digital mental health and social and health care. The European Platform to Promote Well-being and Health in the Workplace (EMPOWER) is the first multi-country project that will involve a maxi impact analysis involving multiple domains to test the use of both the initiation and maturity phases of the GIAF model. (page 3).<br><br>The sentence referred to relates to the ontoterminology approach, not where the domains of GIAF have been used: The ontoterminology approach used in the development of the GIAF methodology as demonstrated in health services research (38).<br><br>The following has been added to strengths and limitations: *A maxi impact assessment using the GIAF framework and toolkit have not previously been used to evaluate an intervention conducted in workplaces, nor across different countries.*
Introduction<br>Page 4, line 18: Please be more specific with regard to describing the topic of this	Abstract aim changed to the following: The aim of this paper is to: describe the methods that will be used to conduct a maxi impact analysis

manuscript. It is the presentation of the methods used for the impact analysis – not the impact analysis itself.	of the EMPOWER platform using the GIAF methodology.
Page 4 Objective and aims<br>Also, in this part it seems like the objective and aims of the impact analysis are too much in focus, rather than the aim of a protocol paper explaining how this will be done (the methods). Instead of highlighting the research questions for the impact analysis, I suggest to highlight more the aims of the protocol paper, which are to show how these aspects can be measured in order to be able to give reliable and informative answers these research questions.	The objectives have been changed to focus on the aim of the protocol paper as suggested. (page 3)
Page 6, line 51: The authors write "The GIAF defines profile as a visual representation that synthesises the information related to relevant domains of a construct or entity. Typically, a profile represents a series of sub-domains of a main entity that cannot be arranged in an ordinal scale or aggregated." Would it be possible to give a visual example of such a profile? The description remains very abstract and it would be good to see an example of such a visual representation of a profile.	The profiles are not relevant to the protocol, rather a representation when we discuss results. We have deleted the reference to profiles to avoid confusion.<br><br>.
Page 6-7, the paragraph about the GIAF checklists: It would be very informative to show an example of such a checklist to get a better understanding for what actually will be measured and assessed.	The checklists are lengthy documents, for example the planning checklist is 10 pages. Each checklist contains a glossary of terms and instructions, and each level of a ladder or profile has the characteristics of that sub-domain, descriptions and examples, and level completion criteria. The checklist also provides space for the rater to document the evidence they have used to rate.<br>An example of one level of the Planning checklist has been added (Table 1, page 7).
Page 7, section about "Implementation sites and target audience"<br>I do not understand what is meant with "Three target audiences will be considered" – considered for what? The intervention? And why is the Empower consortium (who developed the intervention and will evaluate it) also a target audience for the intervention? Please explain.	This sentence has been changed to two target audiences and the EMPOWER consortium and the country coordinators have been deleted.
In the second paragraph in this section the inclusion criteria for the individual employees are explained in detail. However, no information about the inclusion criteria for the	The following information has been added:<br>*Companies will be selected based on being a small or medium-sized enterprise (SME) or public agency (< 250 employees). The Country Coordinators will approach companies who*

workplaces is given and also no information about what kind of information are assessed from the employers (secondary target for the intervention).	*meet the inclusion criteria, provide information about the study and EMPOWER and invite them to participate. Those who consent will enter into a contract between the researchers and employers to affirm ethical considerations, data collection and communications. Each workplace (and thus the employees) will be randomised into the RCT.*<br><br>Table 2 shows the kind of information gathered and assessed from employers: Employer representatives - Standardised questions by the country coordinators via a semi-structured interviews and/or survey with key workplace personnel. Also EMPOWER website usage.
Please also add some information about, what the intervention actually consists of. Otherwise, it remains very abstract what employees and employers/workplaces should be ready for, what they should use, disseminate and adopt. By adding some information about these aspects it will be possible to describe how the measurements will be applied to the EMPOWER intervention.	Section 2.2 has been added and provides an overview of the EMPOWER app for employees and the EMPOWER website for employers.
Moreover, please add some information about the type of workplaces participating in the EMPOWER intervention. Do they come from certain sectors?	The companies are not from any particular sector. The following information was added: *The EMPOWER platform is implemented in small-medium enterprises and public companies <u>from a variety of sectors</u> in three cities and regions in Europe: Barcelona (Catalonia, Spain), Turku (Finland), Lodz (Poland).*
Page 7, section about "Training and preparation for data collection". Please explain who the Country Coordinators and the Country Coordinator teams are. Are they part of the research team?	The following information has been added: *Country coordinators are researchers and part of the EMPOWER consortium. They have been designated in each country (Finland, Poland and Spain) and have attended training. Their tasks include monitoring and managing the trial fieldwork in their country, data entry and validation, solving problems during the fieldwork, and constant communication with the research team.*
Page 8, section about "Data collection methods" The authors write: "The GIAF methodology uses an approach involving the checklists and which use diverse methods of data collection and from multiple sources including end users, employers, Country Coordinators and other consortium groups involved in reporting and dissemination. For each domain in the Initiation and Maturity	This sentence has been re-written for clarity: The GIAF methodology uses an approach involving the checklists and which use diverse methods of data collection and from multiple sources including end users (employees of companies), employers, Country Coordinators and other consortium groups ((e.g., development and implementation groups)

phase, the data collection is via a combination of the following sources of information (the Phase and domain where this method is used is in brackets):"<br>Please explain: Who are the "end users"? What "other consortium groups" are meant here? What is their role and what kind of data do they provide?	involved in reporting development progress and dissemination plans and activities.
The list that follows this paragraph is difficult to understand. Could these information be given in a more informative table where it becomes more clear who is asked about what – referring to the domains from the GIAF framework that were described in detail before? As the list is now, it is difficult to see how this relates to the data collection with regard to the GIAF toolkit.	This information has been combined into a table, which clearly shows who is asked about what, and the GIAF phase and domain/s it relates to (Table 2).
Page 9, line 7: The authors write "There is slightly different wording for the usability questions to the employer, because of their perspective (e.g., budget, available resources)." Does that mean that you adjust the questionnaire to the employers according to their perspective? Please explain.	Sentence changed to the following: There will be slightly different wording for the usability questions to the employer, because of their context and level of knowledge (e.g., budget, available resources).
Page 9, line 8. The authors write: "The name of the workplace (not the representative's name) and position of the responder in the company (e.g., employer, employed manager, supervisor, work health and safety etc.) will be made known to the UC team." Please add why you need to know the name of the workplace. (Also, it should be "work health and safety representative".)	The following sentence was added: *The name of the workplace is necessary so the number of employees in each workplace is known and penetration (the uptake by employees) of the intervention (EMPOWER platform) can be calculated.*<br><br>Representative was added as suggested.
Page 9 section about "Participant and Public Involvement Statement" I do not understand the purpose of this statement at this point of the manuscript. This seems to be something that should have been said much earlier when introducing the study and how the intervention was developed. Please move or integrate better.	We understood this statement is a requirement of the journal.<br><br>This section has been moved to the introduction. (page 3)
Page 9, Section about Organizational learning Considering that organizational learning is the topic of two of the three aims of the study presented on page 4 under Objective and aims, this section is very short and not very precise compared to the many pages that were used to describe the assessment using the GIAF toolkit. The methods used for assessing organizational learning are described on a very broad level with references to many data sources that have not been introduced	The organisational learnings referred to here relate to the Impact Analysis, not the information gained from other studies of EMPOWER.<br><br>The following information has been added: *In this context, organisational learning refers to learnings for the EMPOWER consortium about the EMPOWER intervention and its development and implementation, arising from the Impact Analysis. We will utilise all*

before.	*information gained from each of the Initiation and Maturity domains from each country and synthesise it to generate new insights across the three countries which has the potential to inform and improve future implementation processes. (page 8)*
In addition, it is not clear to me what actually is meant by "organizational learning". It seems not to be the learning that might take place in the participating workplaces/organizations, but rather the learning in the EMPOWER consortium about the EMPOWER intervention (see for example page 9, line 29-30: "We aim to describe the impacts of the EMPOWER project, and provide insights into what worked, in what context and why.")	The following sentence has been added for clarity:<br><br>In this context, organisational learning refers to learnings for the EMPOWER consortium about the EMPOWER intervention.
The methods used for the analysis of organizational learning are also not mentioned in the abstract. Please explain what role the measurement of organizational learning plays in the impact analysis and adjust the level of detail in describing the methods to assess organizational learning accordingly.	The organisational learnings arise from the Impact Analysis – from the data collection through to the results. The learnings will potentially relate to the development and implementation of mental health-e-health interventions in the workplace (the what worked, where, why and how).<br><br>The following sentence was added to the methods in the abstract: These key learnings will be synthesised to generate organisational learnings and insights for the EMPOWER consortium to improve future mental health e-health interventions and implementation processes at the workplace.
Page 9, section about Analysis<br>Similarly, this section is rather short and only very broadly describes how the collected data will be analyzed. For example, I assume there is also a need to translate the answers from Finland, Poland and Spain into English before the four raters from the UC team can rate them for the various domains.	Further information has been added to this section including translation of responses.
Does the last sentence of this section "Both qualitative and quantitative approaches will also highlight key learnings." refer to the analysis of the data collected to assess organizational learnings? Please provide a more detailed description of how the variety of data that was mentioned in the section 2.8 about organizational learnings will be analyzed.	An example has been provided to show how qualitative and quantitative data can inform key learnings.<br>The example provided in the text is:<br>*For example, quantitative data from the usability questions will be compared with qualitative adoption responses and quantitative penetration calculations. This will provide*

	important information such as the extent to which employees in each country considered EMPOWER useful, relevant and of value. This measurement of usefulness may positively influence the degree that EMPOWER is adopted and the extent of the penetration into the target audiences. Country level barriers and facilitators (from Country Coordinator interviews and information from the realist evaluation (employee and employer feedback) will provide context for high or low ratings.
Page 10, Discussion<br>The authors write (line 27) "The EMPOWER project impact analysis will evaluate the process of the implementation; 'how' the EMPOWER intervention was implemented in three countries (Finland, Poland, Spain)"<br>I think it could be added "and to what extend" (after 'how')<br>The sentence continues with : ", and the impacts on both primary and secondary target audiences. The overall EMPOWER project's impacts will also be evaluated." Please explain what is meant by the "overall impact".	'and to what extent' has been added after 'how' as suggested.<br><br>This sentence was changed to the following: *The overall EMPOWER project's impacts, from the development and implementation across the three countries, will also be evaluated*.
Minor comments<br>There are inconsistencies with regard to the numbering of sections: First sections are numbered, but then there is a gap between 2.1 and 2.8 (numbers are missing) and after the section 2.9 the sections are no longer numbered. While this might be less important for the introduction and discussion part, the method section (nr. 2) is very long and would gain from a structure that clearly distinguishes between main sections and relating subsections.	Numbering has been added to each section/ sub-section.
Page 6, line 38: Please check the sentence: "Readiness, dissemination and adoption, dissemination follow..." The "and" seems to be misplaced.	The second instance of dissemination was removed from the sentence.
Page 6, line 51: "The GIAF defines profile as…" (should be profiles)	Changed to, The GIAF defines a profile as…
Page 8, line 12 "The Country Coordinators will also ask the employers the adoption domain questions at the same as the usability questions." The word "time" seems to be missing after "same"	Time added to sentence.
Page 9, line 57: "If differences occur between raters in the level on the ladder/scale/profile,	Changed to 'until a consensus is reached'

the ratings are discussed amongst the UC team, and a consensus reached for the final rating of the project on each domain." I assume you meant to write "until a consensus is reached".	
Page 10, line 11 : "There are complexities with impact analysis of implementation studies such as EMPOWER project with multiple components and the interactions to consider." Add "the" before EMPOWER project.	'The' has been added before EMPOWER Project.
**Reviewer 3**	
1. It is mentioned EMPOWER peoject was "conducted by a consortium of 15 organisations across nine countries", eight of them are european, but only three countries were included in this study. Was there any particular reason?	The following was added to section 2.3: *Three European countries were selected to represent different European welfare and health service models: Finland (represents the Scandinavian model), Spain (Mediterranean model), Poland (Eastern European model).*
2. For the target audiences, why were "members of the EMPOWER consortium including the local country coordinators" included? They obviously have different knowledgement towards this project and should not be compared with other participants. Or were they included here because they were interviewed with other particular questions?	This sentence has been changed to two target audiences and the EMPOWER consortium and the country coordinators have been deleted.
3. It was stated that the local country coordinators translated the questions, will they also translate the qualitative results? Are there any bilingual researcher in the UC team? Could there be any cultural factors that may caused bias?	All the Country coordinators are bilingual in the relevant country. Cultural factors were considered in the translation of the checklist questions from English to (Spanish, Finnish and Polish) e.g. a different word was used as the direct translation into Polish meant something else (p. 9)<br><br>The following has been added to the data collection methods section: Survey and interview responses and Dissemination spreadsheets were written by the Country Coordinators in English which was then provided to UC impact analysis team.
4. It was mentioned "The usability and adoption questionnaires (for both employees and employers) are conducted through a semi-structured interview with the employer representatives.". Who are the employer representatives? Throughout the study, could there be any pressure from the employer that might bias the results of the employee participants?	Examples of employer representatives are provided on page 11: e.g., manager, supervisor, work health and safety representative. 'Both employees and' has been deleted in reference to semi structured interviews. Only employer representatives will be interviewed.<br><br>Employee participants complete anonymous surveys about usefulness which should negate

	any pressure from the employer that may contribute to bias.

VERSION 2 - REVIEW

Reviewer 1

Name Aust, Birgit

Affiliation Det Nationale Forskningscenter for Arbejdsmiljø

Date 04-Mar-2025

COI

The authors have responded to all my comments in a good way and I have no further comments to the revised version of the manuscript.

Reviewer 3

Name Sun, Sijin

Affiliation Imperial College London, Dyson School of Design Engineering

Date 09-Mar-2025

COI

My comments from the first round of reviewing have been sufficiently addressed. I hope the adjustments made in the protocol will be followed in the delivery of actual research.
